# Effects in spite of tough constraints - A theory of change based investigation of contextual and implementation factors affecting the results of a performance based financing scheme extended to malnutrition in Burundi

**DOI:** 10.1371/journal.pone.0226376

**Published:** 2020-01-13

**Authors:** Manassé Nimpagaritse, Catherine Korachais, Bruno Meessen

**Affiliations:** 1 Institut National de Santé Publique, Bujumbura, Burundi; 2 Health Economics Unit, Department of Public Health, Institute of Tropical Medicine, Antwerp, Belgium; 3 Institut de Recherche Santé et Société, Université Catholique de Louvain, Clos Chapelle-aux-Champs, Bruxelles, Belgique; BITS Pilani, INDIA

## Abstract

**Background:**

From January 2015 to December 2016, the health authorities in Burundi piloted the inclusion of child nutrition services into the pre-existing performance-based financing free health care policy (PBF-FHC). An impact evaluation, focused on health centres, found positive effects both in terms of volume of services and quality of care. To some extent, this result is puzzling given the harshness of the contextual constraints related to the fragile setting.

**Methods:**

With a multi-methods approach, we explored how contextual and implementation constraints interacted with the pre-identified tracks of effect transmission embodied in the intervention. For our analysis, we used a hypothetical Theory of Change (ToC) that mapped a set of seven tracks through which the intervention might develop positive effects for children suffering from malnutrition. We built our analysis on (1) findings from the facility surveys and (2) extra qualitative data (logbooks, interviews and operational document reviews).

**Findings:**

Our results suggest that six constraints have weighted upon the intervention: (1) initial low skills of health workers; (2) unavailability of resources (including nutritional dietary inputs and equipment); (3) payment delays; (4) suboptimal information; (5) restrictions on autonomy; and (6) low intensity of supervision. Together, they have affected the intensity of the intervention, especially during its first year. From our analysis of the ToC, we noted that the positive effects largely occurred as a result of the incentive and information tracks. Qualitative data suggests that health centres have circumvented the many constraints by relying on a community-based recruitment strategy and a better management of inputs at the level of the facility and the patient himself.

**Conclusion:**

Frontline actors have agency: when incentives are right, they take the initiative and find solutions. However, they cannot perform miracles: Burundi needs a holistic societal strategy to resolve the structural problem of child malnutrition.

**Trial registration:**

Clinical Trials.gov Identifier: NCT02721160; March 2016 (retrospectively registered).

## Background

Performance-Based Financing (PBF) schemes and policies are increasingly being implemented in low income countries (LIC) to improve population health through better health service delivery and stronger health systems. The core mechanism of PBF is the payment contract, which incentivises healthcare facilities to better align their service provision with population and health system interests [1].

Whilst an appropriate payment structure is essential for the success of PBF schemes, it is acknowledged today that the theory of change (ToC) of the strategy is richer than that [1,2]. Most PBF schemes are complex packages of interventions including components such as strengthening of performance monitoring and feedback systems, greater autonomy for health facilities, digitalisation of health performance data, or capacity building measures [3,4]. All of these can also impact staff behaviours and the performance of health facilities [5].

Seeing PBF as an intervention which rearranges the pre-existing ‘nexus of institutions’ [6,7], with a more or less explicit ambition of reform [8] entails two things. Firstly, to the extent that an institution is a shared pattern of social behaviours [9], it is the actual enactment of the scheme which matters. Secondly, analysts and researchers must apprehend a PBF intervention within its context. The latter indeed comprises a lot of institutions and other drivers of change that may affect the nature of the scheme and its effects. Context matters at the adoption stage (with some tailoring of the package to the setting, needs, opportunities, for example), but also during implementation: a supportive context (e.g. the availability of medicines) will enhance the effectiveness of the strategy and, probably, its sustainability.

This view of PBF as an institutional intervention implemented in a ‘complex adaptive system’ [10] has gained considerable traction within the research community in recent years. Alongside evaluations focused on the impact of PBF [11–13], we have now studies investigating the actual theories of change, with a plurality of situations and practices which prevail. One can highlight, for instance, the recent work by the ReBUILD team on how PBF works in Fragile and post-Conflict Affected Settings (FCAS) [14–16], the comprehensive analysis of the RBF scheme in Malawi by De Allegri and colleagues [17] or the research program carried out by Borghi and colleagues, mainly in Tanzania [18].

Our study in Burundi belongs to this program of understanding change mechanisms triggered by PBF in a specific context and documenting how they affect the outcome of the strategy.

In this paper, we have focussed on the introduction of malnutrition prevention and care ‘indicators’ within the pre-existing PBF-free health care program in Burundi. We have worked to advance our understanding of the contextual and implementation factors that influenced the course of the intervention and its outcomes. More particularly, we checked how these factors interacted with the pre-identified tracks of effect transmission embodied in the intervention. This case is interesting for at least three reasons: (1) it is about an ‘update’ of a pre-existing PBF program (integration of new services for remuneration by the scheme); (2) this update is about a health problem that is particularly difficult to address (child malnutrition); and (3) it is in a country which, during the course of the intervention, moved from relative stability to greater fragility.

We first provide some general information on the context in Burundi; we then give an overview of the initial ToC, our analytical approach and the data. The findings of our study are then presented in four steps: (i) information on factors which have affected the intervention, (ii) the activation or not of the different tracks of the ToC, (iii) the possible routes to obtaining positive results, and (iv) an updated ToC. The article concludes with a discussion of our findings and some directions for policymakers and researchers.

### Context (before the intervention)

Burundi is a small landlocked country in eastern Central Africa. Since its independence, Burundi has experienced continuing waves of instability; from 1993, the country was in a state of civil war which ended with the Arusha agreements, signed in 2000. The elections organised in 2005 allowed the country to return to stability and security until April 2015, when it again plunged into a political crisis. Among the 178 countries assessed under the Fragile States Index 2016, Burundi ranked as the 15^th^ most fragile country [19].

Burundi is one of the World’s poorest countries, with a GDP per capita estimated in 2016 at PPP $ 777 [20]. Its economy is heavily reliant on the subsistence agricultural sector, which employs more than 80% of the population. Export is very limited and the main source of currencies is aid [21]. With a total population of 10.2 million, Burundi has a high population density (397 inhabitants per km squared, 2015) and continues to have a high synthetic fertility index (5.8 children per woman, 2015) [22]. At the time of the Arusha peace agreement, the health status of the population was amongst the worst in the World. Since then, the government of Burundi and its partners have been investing in the health sector and significant progress has been made. One axis has been the reorganisation of the health system into health districts [23]. The health centre is the point of entry into the health care system with a minimum package of activities to be offered, including prevention and treatment for malnutrition.

In 2006, the government removed user fees for children under five and deliveries in all health facilities [24]. In parallel, Burundi, inspired by neighbouring Rwanda, had started to pilot a PBF strategy in three provinces [25–27]. In 2010, the PBF strategy–whose core proposition is to remunerate health facilities along performance measures capturing both volume and quality aspects–was scaled up nationwide and merged with the selective free health care strategy [28].

The PBF-Free Health Care (PBF-FHC) policy finances a quite broad package of services, with a focus on maternal and child health. In spite of a high prevalence of global acute malnutrition (6% in 2010) [29], the initial scheme did not cover nutrition services. This was in line with practice in other early PBF schemes [6]: PBF is a supply-side strategy focusing on health facilities only and given the multi-causal nature of malnutrition, designers of the PBF-FHC saw malnutrition as a tough nut to crack.

In the following years, several studies confirmed the effectiveness of the national PBF-FHC policy [30–34]. This gave confidence to health authorities and in 2015, with the support of the World Bank, they piloted the inclusion of nutrition services into the pre-existing PBF-FHC scheme at three levels–hospital, health centre and community–on both prevention and care aspects, all focusing on children below five years old. This consisted in adding nutrition indicators (on both prevention and care aspects, with a focus on children below five years old) to the existing performance grid, for the sample of pilot health facilities.

## Methods

### Study design

The introduction of nutrition services into the PBF scheme (hereafter referred as PBF-N or “the intervention”, see S1 File for a presentation of the reward structure) was piloted from January 2015 to December 2016, with a rigorous impact evaluation. The research design was a cluster randomised control trial, whose main aim was to measure the effects of the PBF-N on nutrition outcomes (45 intervention HCs received a payment conditional to their performance on a list of nutrition indicators, 45 control HCs received a financial compensation equivalent to the PBF subsidies received on average by intervention HCs). A full presentation of the intervention and the research design and an introduction to the various research instruments are available in Nimpagaritse et al. [35].

As reported in the study report [36] and in a forthcoming paper [37], findings of the impact evaluation are actually mixed. As highlighted in Table 1 below, several important indicators (such as the number of Moderate Acute Malnutrition (MAM) and Severe Acute Malnutrition (SAM) cases identified and managed, MAM recovery rates, MAM and SAM duration of treatment) have improved thanks to the PBF-N. The PBF-N led to an increase of volume combined with an improvement in outcomes at the facility level. However, this did not translate into changes in the prevalence of malnutrition at the community level and on several other metrics at the HC level (growth monitoring, malnutrition screening during curative consultations).

**Table 1:** Impact of the PBF-N on some indicators.

Indicators	Period	Control group		Intervention group		Impact	p-value
		*Mean*	*N*	*Mean*	*N*		
***Improvement of the performance in the intervention group (significant)***							
1. MAM cases over the last six months	Before	62	45	33	45	+138	<0.001
	After	13	45	122	45		
2. SAM cases over the last six months	Before	40	45	25	45	+53	<0.001
	After	42	45	79	45		
3.MAM recovery rate over the last six months	Before	76%	308	84%	320	+14.7pp	0.007
	After	78%	112	97%	327		
4.Treatment duration among MAM cases (days) over the last six months	Before	71	225	78	252	-29.3	0.047
	After	70	77	44	288		
5.Treatment duration among SAM cases (days) over the last six months	Before	57	304	61	252	-19.5	0.021
	After	59	317	43	282		
***Improvement of the performance in the intervention group but not significant***							
6.Chronic malnutrition prevalence at community level on the day of the household survey	before	53.3%	3100	53.6%	3099	+1.8pp	0.378
	after	49.9%	3234	52.0%	3246		
***Deterioration of the performance but not significant***							
7.Acute malnutrition prevalence at community level on the day of the household survey	before	6.3%	3100	5.8%	3099	+0.5pp	0.634
	after	8.8%	3234	8.7%	3246		
8.Growth curve mentioned during the consultation on the day of the facility survey (observation of consultation)	before	6.2%	260	8.3%	254	-1.11pp	0.763
	after	3.8%	263	4.2%	265		
9.Growth monitoring sessions regularly organised over the last six months	before	80%	45	84.4%	45	+14.8pp	0.152
	after	51.1%	45	77.8%	45		
10.Growth curve displayed in health booklets on the day of the household survey	before	8.1%	2472	12.7%	2445	+6.5pp	0.214
	after	1.2%	2946	4.6%	2941		
11.False positive rate of acute malnutrition diagnosis on the day of the facility survey (exit interview)	before	1.00%	2/205	1.33%	3/226	+1.62pp	0.639
	after	0.45%	1/223	1.80%	4/260		
12.False negative rate of acute malnutrition on the day of the facility survey (exit interview)	before	86.5%	45/52	92.6%	25/27	-0.26pp	0.983
	after	80,0%	32/40	88.4%	38/43		
13.Anthropometric equipment available and functional on the day of the facility survey	before	11%	36	11%	38	+3.97pp	0.693
	after	4%	45	7%	45		

**Fig 1:**
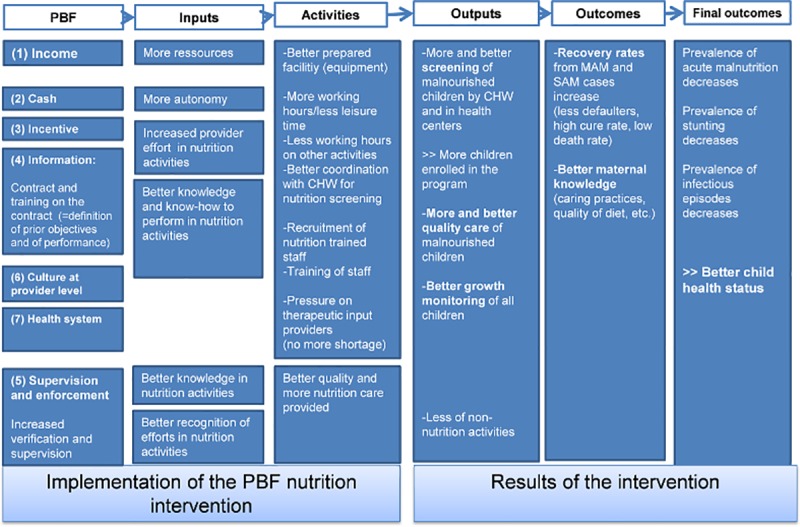
Initial ToC mapping the tracks through which Nutrition PBF affects the health system and results in outcomes.

Aware of the challenges presented by malnutrition, the research group had anticipated the possibility of limited impact of the PBF-N. At the protocol development stage, attention had been put in an extensive mapping of tracks through which the PBF-N intervention might generate effects for children suffering from malnutrition (see Fig 1). Some of these tracks are well-known as central to the ToC of PBF (e.g. the ‘incentive track’), some are more speculative (e.g. the ‘supervision track’) and were listed so as not to miss something. This theoretical mapping inspired the development of a large set of research instruments to capture the transmission (or non-transmission) of the effects of the PBF scheme at the HC level [35]. We will more extensively present and use these seven tracks to structure our findings in the results section.

The aim of this paper is to generate a better understanding of the reasons why the PBF intervention had mixed effects on our primary malnutrition variables measured at the health facility level. We see two main questions to investigate empirically: (i) which constraints have had an impact on the transmission of the effect of the PBF-N; (ii) through which routes the PBF-N had still some effects on some output (column 4 in Fig 1) and outcome variables (column 5 in Fig 1).

We largely built our analysis on (i) findings from the quantitative impact evaluation (surveys performed over September-October 2014 for the baseline, and February 2017 for the follow-up–with findings sometimes already reported in other publications) and (ii) extra qualitative data. The qualitative data mainly exists within logbooks filled in by HC staff and in-depth interviews with key informants from HCs and Health District Management Teams (HDMT). When relevant, we have also added some extra information from PBF-N routine data and PBF-FHC annual reports.

The logbooks were to be filled in on a weekly basis by the HC managers during the whole period of the intervention; they had been invited to document the nutrition activities, inside the health facility, with the Community Health Workers (CHWs), and in the catchment area of the HC. In their logbooks, HC managers had to report some basic information in regard to nutrition services as well as (i) bottlenecks (e.g. a stockout of nutritional inputs), (ii) extra financial or in-kind assistance received (from technical and financial partners such as NGOs) and (iii) initiatives taken by the HC staff to improve the performance of the nutrition services (e.g. meeting with CHWs to improve the retrieval of children lost in follow-up). In each province, twice over the two years of the intervention, the 1^st^ author (MN) facilitated a workshop around these logbooks with HC managers. Seventy five out the ninety HC managers completely filled in well their logbooks. The reported data were transcribed into an excel sheet for analysis.

Twelve out of the 90 HCs covered by the impact evaluation, including public and private non-for-profit HCs, were purposively selected for the key informant interviews. They were selected within each group (six HCs from the intervention group and six HCs from the control group) depending on the changes observed through the quantitative impact evaluation in nutrition performance with regards to MAM and SAM recovery rates and duration of treatment. Interviewees were purposefully chosen to reflect a mix of HC managers (n = 12), nutrition service managers (n = 12) and those who acted as the focal person for nutrition services of HDMT (n = 8).

Based on the initial ToC, a semi-structured interview guide was prepared by the lead investigator (MN, who is a Burundian medical doctor with complementary international training in health system and qualitative research). The interview was pilot-tested in order to set up all the materials and try out the whole procedure before running the interviews with real participants.

Participants were contacted and briefed via telephone; interviews were carried out from March to May 2018.

The interviews were conducted in Kirundi and tape recorded, except for two respondents who refused to be recorded and for whom we considered the notes taken during their interviews. All interviews took place in a private room at the health center.

Audio tapes were transcribed in the original language. For both data (logbooks and interviews), data analysis was performed using a framework approach [38] based on a series of predefined codes which reflected the main elements of the initial ToC. The analysis followed the five stages of (i) familiarization with data, (ii) identification of themes, (iii) indexing, (iv) labeling and (v) mapping and interpretation. Verbatim reporting in this paper has been translated by the 1^st^ author. At the analytical stage, we performed our analysis using an "average/general/majority effects approach" first and foremost. Possible heterogeneity among health centres is mentioned only where really obvious.

We applied data triangulation by comparing findings across data sources (interviews and logbooks) and also across respondents (HDMT and HC manager and provider). Furthermore, when needed, we referred to data from other sources (impact evaluation, PBF-FHC annual reports and PBF-N routine data), to advance our understanding of the mechanisms.

## Results

Our results section is organised in four parts.

In a first section, we provide important information regarding the environment in which the PBF-N was embedded, the pre-existing constraints at HCs level and the implementation issues. PBF-N was expected to empower existing interventions (mainly nutrition services at HC level) and build upon some key ancillary functions of the whole health system. As we shall see, serious problems appear at these levels.

In the second section, we review the implementation of the PBF-N using our seven-track framework (Fig 1). We map both the constraints and the enablers which have affected the intervention. For each track, we remind first how it was expected to contribute to some effects and then we assessed whether the track was actually activated or not.

Our third section is a bit more speculative. It addresses our second research question: we try to understand how despite the many constraints presented in sections 1 and 2, the PBF-N still generated some effects.

Our fourth section acts as a synthesis: we put forward a revised version of the ToC of the PBF-N.

### The environment embedding the PBF-N

**Fig 2:**
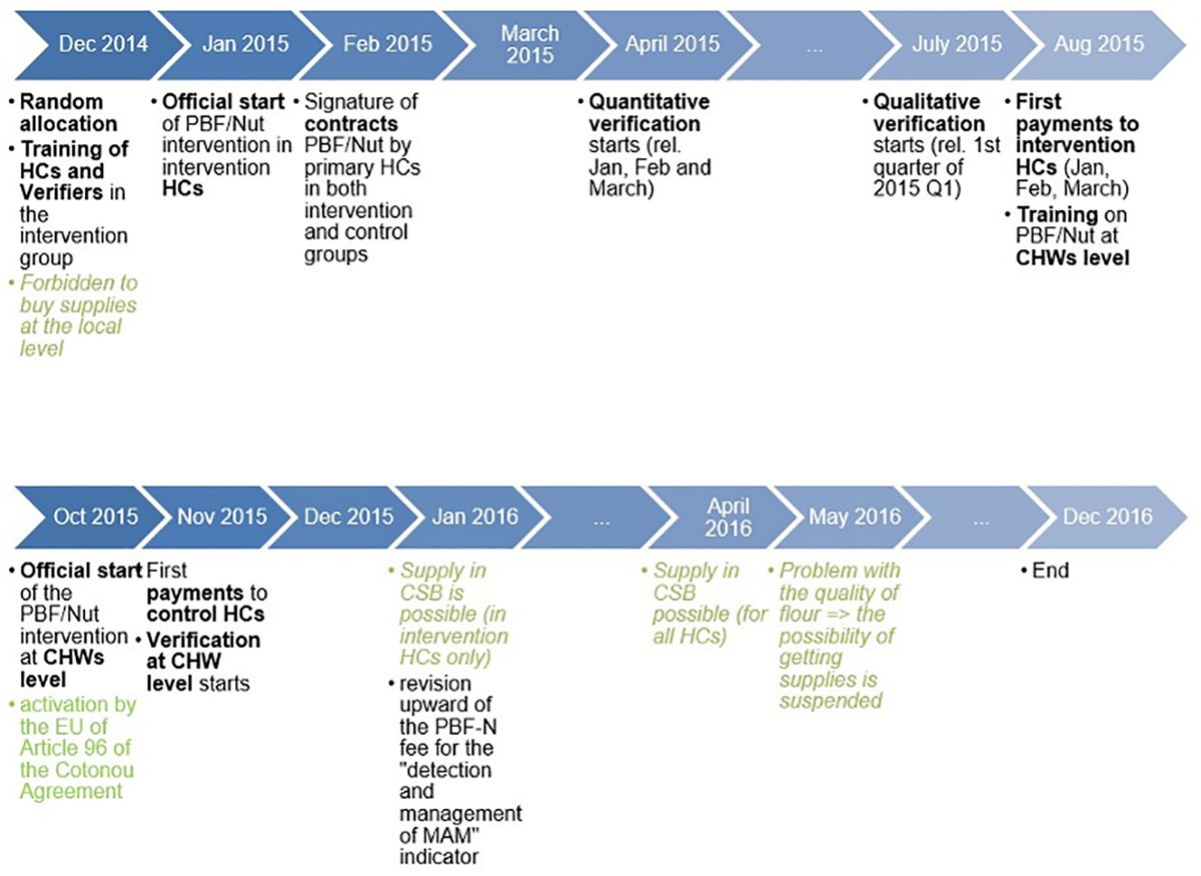
Timeline of PBF-N implementation.

The overall environment has been unfavourable to the intervention. There have been major problems with the provision of nutritional supplements for nearly the whole duration of the intervention. Problems already arose before the start of the intervention. Indeed, a bad hit (not foreseen at protocol stage) was the decision of WFP in 2014 to stop supplying nutritional supplements (Corn Soya Blend-CSB- + sugar + oil) in all provinces but two. To mitigate the consequences, it was decided on October 2014 to adapt the intervention and revise upward the PBF-N fee for the "detection and management of MAM" indicator up to an amount which was hoped to allow HCs to purchase these supplements locally (a solution fully in line with the standard recommendation under PBF programs to allow health facilities to choose their suppliers). However, a few days before the start of the intervention, the nutrition program of the MoH (*PRONIANUT*) changed its mind and did not permit decentralised purchases by each HC. The lack of training of HC managers in agri-food technology and the impossibility for *PRONIANUT* to manage quality control were cited as reasons for this change. This was a blow for the intervention, as it meant that HCs would be expected to expand their nutritional services for moderate acute malnourished children without using food supplements. This problem had an impact on the intervention until December 2015, as highlighted in our timeline of the intervention (Fig 2). The solution put forward by *PRONIANUT* was to start a procurement process to identify and select one single supplier for the whole country. However, the administrative process took a whole year. In December 2015, a local flour producer was chosen. In January 2016, it was, at last, possible for intervention HCs to order the food supplement and receive the first deliveries. Orders from HCs and deliveries to the latter were made through health district offices on a quarterly basis (as we shall later see, this led to other problems). The control HCs could have their first deliveries in April 2016 (but only 12 of them made supplement orders in 2016).

However the problems were not over. In May 2016, quality problems with the flours were reported. Consequently, supply from the local producer was temporarily suspended and only started again in September 2016, just three months before the end of the intervention in December 2016.

A second shock arose that was external to the health sector, but also related to the aid sector. In April 2015, a socio-political crisis erupted in the country [20]. This led some donors, including those participating in the financing of the PBF-FHC, to review their modes of cooperation with Burundi. The European Union and European countries activated Article 96 of the Cotonou Agreement in October 2015 [39]. This led to the interruption of several aid programs and the institutional reorganisation of others. As we shall see in section 2, this situation resulted in financing gaps and arrears for health financing schemes (PBF-FHC and *Carte d’Assistance Médicale*-*CAM*) crucial for the economy of HCs.

### Constraints at facility level

There were also constraints at a HC level. We have a clear picture of those as a result of the quantitative baseline survey [40].

The first constraint relates to the quantity and quality of health staff for the management of malnourished children. As shown in Table 2, there is a general pattern of low initial education, lack of supervision and lack of in-service training.

**Table 2:** Training and supervision of HWs at HC level (September 2014).

	Cases (%)
***Degrees of HWs among all employees of the HCs (90 HCs surveyed)***	
HWs with an A3 degree (2 years of nursing training at secondary school)	25 (28%)
HWs with an A2 degree (4 years of nursing training at secondary school)	19 (21%)
HWs with an A1 degree (2 years of nursing training at university) or higher	1 (1%)
***Distribution of HWs by degree by HCs***	
HCs without HWs with level A1 degree	78 (87%)
HCs without HWs with levels A1 degree or A2	5 (6%)
Compliance with staff norms of a HC in Burundi*	
HCs with three or more nurses with an A2 degree (or higher)	58 (64%)
HCs with three or more nurses with an A3 degree	69 (77%)
HCs with a health promotion technician	46 (51%)
***Supervision of nutrition services***	
HCs with nutrition services visited by the district supervision team over the last six months	40 (44%)
HCs with nutrition services visited by the district nutrition focal point (n = 40) over the last six months	22 (55%)
***Continuing professional education of HWs (n = 145) after their initial training***	
Received training on growth monitoring and promotion	21 (15%)
Received training on screening and management of acute malnutrition	60 (42%)
Received training on management of complicated SAM	33 (23%)

(*) at least three nurses with an A2 diploma (or higher), three nurses with an A3 diploma, one lab technician, one health promotion technician and one financial manager

This limited human resource capacity, which is a standard pattern in rural Burundi, was an important constraint on the likelihood of delivering quality nutrition services. The baseline study shows that during curative consultations, only a small proportion of children were interrogated about their diet and few HWs consistently asked about four (maximum) out of the seven questions relating to malnutrition. In addition, most of the children missed the opportunity of having accurate nutritional assessment and detection of malnutrition with anthropometric measurements, for further details see [41].

A second constraint has been the availability of both equipment for nutritional services and systematic as well as food treatments. At baseline, only 14% HCs had all the equipment and tools (available and functional) needed for the screening of children for malnutrition. Overall, only 24% had the required systematic medical treatment for malnutrition services [40].

### Problems with the implementation of the intervention

The intervention started in January 2015 (see timeline, Fig 2). HC managers of the intervention group had been trained in the PBF-N scheme during a one-day workshop in early December 2014. The signature of contracts by HCs was delayed by one month and was effective from February 2015. These contracts were the existing PBF-FHC ones, adapted by adding a specific article relating to the PBF-N intervention.

During the whole intervention, the verification system used was roughly the same as for other PBF-FHC. Health centres report monthly on the quantity of incentivised services delivered. These reports were verified and validated at the provincial level by the Provincial Committee of Verification and Validation (*CPVV* in French) and sent to the ‘*Cellule Technique Nationale du Financement Basé sur la Performance’*—CTN-FBP—(the MoH unit in charge of the PBF-FHC at a national level), which had to approve transfers of subsidies to the HCs. In addition, a qualitative assessment (checklist) was completed quarterly.

Quantitative verification of the PBF-N began in April 2015, targeting performance data from the three previous months. It was carried out monthly thereafter.

The first qualitative assessment took place at the end of July 2015 and covered the performance data of the first two quarters of 2015. It appeared that for many HCs, the quality scores were very low. The *CTN-FBP* took the exceptional decision not to take them into account for the calculation of the first PBF-N funding for the HCs. This decision was motivated by the fact that, because of the penalty system (for the reward system, see S1 File), many HCs would have not received any funding from the PBF-N. The scores of the qualitative verification began to be used in the calculation of the PBF-N funding only with the assessment of the performance data of the third quarter of 2015.

As for the PBF-FHC, PBF-N subsidies were transferred to the bank accounts of each HC. The first transfer began 8 months after the start of the intervention. The control group HCs received their first payments at the end of November 2015.

### Analysis of the implementation of the intervention

In the following section, we analyse how all these constraints affected the different pathways/tracks through which effects were expected to happen. Information emerging from the data is assessed at the end of each track.

#### The income track

The ‘income track’ refers to the fact that part of the effect of the PBF-N scheme may come from the increase in revenue. Greater resources allow HCs to provide more and better services.

Management data from the *CTN-FBP* shows that, on average, HCs received around 1,300,000 BIF (830 USD) from the PBF-N in 2015 and around 30,000,000 BIF (17,750 USD) in 2016. Note that the amount granted in 2016 included the money required to purchase dietary inputs, via the high fee adopted for the activity ‘management of MAM’ (90% of the fee was estimated to be necessary for the purchase of inputs). Then, on average, in 2015 a HC received less than 70 USD per month. This means that for HCs participating in the pilot study, the revenue from PBF-N corresponded to less than 2% of the total PBF revenue. So, an injection of resources took place, but it only became substantial in 2016, as (i) the fee for MAM cases was increased and (ii) performance improved.

However, this was not without problems. Firstly, as mentioned in our previous section, there were delays in receiving the first PBF-N subsidies. This was at odds with the expectations of health facilities as outlined in the PBF policy, which stated that usually payment of the quantitative benefits of health facilities would occur within 50 working days following the month concerned [42]. We have seen above that a major cause of this delay, the quantitative verification for the first three months of activities, took place in April only (under the PBF-FHC, it takes place each month).

“… *they spent too much time before we had the first payments*, *which led to a decrease in bank reserves…” (HC manager 602I6)*

Secondly, analysis of the logbooks revealed that, during the period of the intervention, all HCs also experienced arrears with the existing PBF-FHC scheme. We have triangulated this information with management data from the *CTN-FBP*; these arrears were around 4.3 billion BIF for the year 2015 and 4.8 billion BIF for the year 2016. The annual PBF-FHC reports of 2015 and 2016 attribute this problem to the public finance crisis induced by the political crisis of 2015, first through the suspension of aid by some partners and then the temporary unavailability of financial resources at a government level [43,44]. These problems affected the daily functioning of some health facilities.

***Our assessment*:** this track was activated, with a rather low intensity at the beginning; by design, its intensity increased along with the improvement in the performance of HCs.

#### The cash track

A key principle of PBF is to pay health facilities directly into their bank account. The ‘cash track’ refers to the fact that part of the effect of the PBF-N scheme may come from this availability of cash at a facility level; it allows the health centre to rapidly and autonomously address any local bottlenecks. They can, for instance, purchase key items (for example, drugs, food, fuel or stationary), hire some manpower or invest in infrastructure (for example, motorbikes or equipment).

As expected, the PBF-N payments were paid into the bank accounts of health facilities. In terms of utilisation, the same rule as the PBF-FHC applied.

Qualitative interviews confirm that the subsidies earned with PBF-N intervention were transferred to the bank account of each HC, lumped together with the subsidies of the pre-existing PBF-FHC. According to some interviewees, this limited visibility (no specific labelling) and the full fungibility with the other financial resources of health facilities may have not encouraged HCs to use PBF-N subsidies first and foremost to improve nutrition services.

“*Subsidies from the PBF-N intervention were paid into the HCs account together with other payments and used to carry out planned activities like all resources of the HC*.” (In-charge of nutritional services 602I6*)*

The PBF system and managerial tools organise the full fungibility of revenues at health facility level.

“*The PBF-N subsidies were helpful because they supported other revenues*, *which allowed the planned activities to be carried out*. *We cannot say that they allowed us to do only the nutrition activities*, *but rather according to the guidelines of the PBF in general*, *as you see*, *our HC is equipped*, *we built a fence*, … *it is in this way that we used them*.*”* (HC manager 1402I5)

In their logbooks, some HC managers complained that they could not easily identify the PBF subsidies in the history of their bank accounts. This traceability problem is due to the practice of some banks of not integrating the communication text in their transfer. This problem is also mentioned in the 2016 annual report of the *CTN-FBP* as one of the constraints of implementation of the PBF-FHC.

A major constraint to the effectiveness of the cash track was, however, the impossibility to purchase food supplements for MAM children. As noted above, this supply was initiated one year after the intervention started. For HCs with cash flow problems, the absence of a credit facility for the purchase of food supplements was another constraint.

“*The Health District office requested payment of the invoice directly upon delivery*, *therefore before we received the PBF nutrition subsidies*.*”* (HC manager 602I6)

***Our assessment***: this track was activated. HCs received the payments into their bank accounts and had control over these resources, fungible with other revenues.

#### The incentive track

By definition, a PBF scheme rewards health facilities according to their performance. The ‘incentive track’ refers to the fact that part of the effect of the PBF-N scheme is expected to come from the conditionality put upon the extra resources. To raise the revenue of the health facility (and possibly their personal income), staff will adopt strategies likely to increase performance as measured by the PBF-N scheme: they may review their priorities and the general allocation of their time, work extra hours, optimise the organisation of services, better comply with guidelines or ensure better management of their stocks, etc.

A requirement for this track to work is for HC staff to be aware of the payment rule, to understand it, observe the link between their performance data and the paid amounts and trust the whole system.

Because of years of presence of the PBF-FHC, the PBF logic is well-known by all HC managers in Burundi.

“*This meant that the HC should take care of cases of malnutrition and receive in return the funding corresponding to cases treated and cured after verification and validation each time by the persons in charge of the verification within the CPVV*.*”* (In-charge of Nutritional services 902I1)

A factor which could have weakened the impact of the incentive track is the fact that the PBF-N ‘indicators’ were inserted into a larger set of pre-existing ‘indicators’, and they did not get a special status in terms of visibility or allocation. As mentioned above, nutrition PBF revenues were lumped with the subsidies of the pre-existing PBF-FHC, payment was disbursed globally and relatively speaking constituted a limited share of the total PBF revenue. The income from the PBF-N was not ear-marked (for instance to be paid to and managed by the nutrition staff). All these rules make sense given the Burundi’s choice for integrated management at the health facility level, but they could have blurred the power of the incentive and left the scheme at the mercy of the overall economic conditions of the health facility or of the PBF-FHC.

Nevertheless, there is evidence that staff responded to the incentive. In general, because of the many constraints, especially the limited technical skills, health facilities scored poorly for the qualitative component, during the whole intervention. Still, routine data from the PBF-N shows an improvement for four of the six dimensions carrying the greatest weight in the quality checklist (dimensions 5 & 6 of Table 3 and dimensions 5 & 6 of Table 4 in S1 File). The annual average scores on the ‘compliance with the performance indicators’ dimension improved for both MAM (from 12.8/30 in 2015 to 18.2/30 in 2016) and SAM services (from 3.0/30 in 2015 to 16.4/30 in 2016). We observe a similar pattern for ‘correct case management’ activity. Though it has stayed at a rather low level for MAM, it has still risen sharply from 3.9/20 in 2015 to 15.1/20 in 2016. This suggests that HC staff paid attention to the incentive structure, even if this did not always lead to significant changes (as measured by the surveys).

At the individual level, a constraint upon the incentive track is that in Burundi, bonuses are distributed to staff only if other costs are covered.

This is highlighted below, as phrased by an In-charge of nutrition services:

“. . . . *the bonus is not regular*, *for example it's been a long time since we got one (*…*) I heard that the health facilities got payment from the PBF-N*. *Actually*, *we cannot see the history of the bank accounts of the HC*. *(…) When a bonus was paid*, *I could not know the origin; so*, *if they said that a bonus was available*, *I took it*, *like other colleagues*.*”* (In-charge of nutrition services 1602C1)

In fact, the years during the intervention have been the most difficult ones since the introduction of the national PBF-FHC in 2010. Since 2015, the government has been struggling in honouring its own financial commitments, for the PBF-FHC, and also for other schemes. The economic situation of health facilities has deteriorated and arrears constrained the payment of bonuses to staff, which is not good for staff morale [43].

Eventually, for the incentive track to work, HC staff must be in a position to solve the problems they identify. The supply problems with the food supplement have generated a lot of frustration. As a professional, one must wonder what is the point in attracting more patients or better detecting malnutrition in children if one lacks a key component to provide appropriate care?

***Our assessment***: this track was activated; despite tight constraints, HWs managed to improve the indicators upon which they had control (see further).

#### The information track

The ‘information track’ refers to the fact that some of the effects of a PBF scheme may come from the extra information conveyed by the intervention to health staff. Key information lies in the contract itself. The list of rewarded indicators provides the health facility manager with a rather clear template on the HC’s roles and responsibilities with regards to nutrition services. Some useful information is also generated by the monitoring process (as each HC is informed about its performance in terms of volume and quality of services).

To have an effect from this track, one needs an intelligible contract, strong signals from the fee system (e.g. absolute and relative weights to say what is important) and good information sessions at the start of the scheme. In addition, continuous feedback from actors involved in the implementation (supervisors from the department for nutrition within the MoH, district supervisors and persons in charge of the verification within the CPVV) may also remind health facility staff what really matters.

As stated above, in the first section, HC managers were trained on the intervention during a one-day workshop. The informational benefit from this training is confirmed by this HC manager:

“*We were very happy with the training we received prior to the start of the intervention because we were shown what we would do next*, *how we were going to do it*, *how the intervention would be implemented*, *and how we were going to ensure the care of our patients*.*” (*HC manager 101I4)

There was, however, some time lapse afterwards; contracts were signed two months following the training. More fundamentally, the slow initiation of the activities affected the effectiveness of the training.

“*Time has elapsed between the training and the beginning of the intervention*, *that's a challenge*. *Ideally*, *each trained person should have applied what he learned*. *It took a while for the intervention to start*. *Then*, *there are things that you do not do right*, *because in the meantime you forgot*.*”* (HC manager101I4)

The more a program uses opportunities to communicate its content to its implementers, the better. The interviews revealed that some opportunities were missed. Some key actors who had to support HCs in relation to the content of the intervention were not sufficiently informed in advance. This is particularly the case of health district supervisors and the persons in charge of the verification of the quantitative component of the scheme.

“. . . . . . . *as I did not attend any meetings related to the intervention*, *I could not judge whether they experienced or not difficulties in implementation* . . . . *I could not distinguish the intervention from the rest*. *I only heard that there had been an early meeting attended by the District Chief Medical Officer and the managers of HCs involved in the impact evaluation; but nothing more*.” (HDMT member 902D)

“*Yes*, *at the beginning it was hard with the quantity verification; the persons in charge of verification even told us that they did not fully understand the indicators to verify*. *We do not know whether they had received training to do verification properly*. *Sometimes*, *they were really confused on what to count*: *either the cured cases or the treated and cured cases*. *Others wanted to count the admitted cases only*.*”* (In-charge of nutrition services 602I6)

We have already reported the delay with the verification process at the initiation of the program. To some extent, this has also affected this track, as verification is an important opportunity to re-explain a PBF contract. Eventually, it is not also sure that despite clear guidance, the verification visits were performed in a way conducive to learning.

“*With this way of verification*, *auditors come in as police officers*, *yes they are real police officers*. *When they arrive at the HC*, *it's as if they are not partners*, *they come as if they are there to give orders or as if they come to point at the errors only*. *They do not take the time to show us exactly what to do*. *It is as if it is to set us a trap* … *Actually*, *when they are there*, *some providers are scared*.*”* (In-charge of nutrition services 602I6)

***Our assessment***: this track was also activated. HWs were aware of the nutrition indicators to be rewarded and their relative weights. However, the slowness in implementing the training content as well as the limited capacities of actors involved in follow-up during implementation have weakened its effectiveness.

#### The ‘culture at provider level’ track

As a policy, PBF comes with a discourse promoting entrepreneurship at the facility level; managers are encouraged to take initiative to boost the performance of the HC. The ‘culture at provider level’ track refers to the fact that part of the effect of a PBF scheme may come from this encouragement of entrepreneurship (independently from the reward system). This track can be identified as a ‘slow one’: it requires the emergence of new aptitudes at both individual and organisational levels and can take months or even years to emerge. Given the fact that PBF-FHC was already in place for 5 years in all HCs, we can assume that this track had already been activated before the PBF-N.

None of our research instruments really tried to capture entrepreneurship capacities at the HC level. It is, therefore, difficult to evidence whether these capacities were strengthened by the intervention or even determine their initial baseline level. We have no qualitative evidence indicating that a special effort in favour of entrepreneurship was carried out by the *CTN* or the *PRONIANUT* during the intervention. Our assessment is that the intervention relied on the entrepreneurial ethos already present in the HCs (if any).

In the logbook, there was a section where HWs had to report initiatives taken to improve the performance of nutrition services. Twenty-two HCs from the intervention group reported some initiatives.

From the logbooks and our interviews, it appears that initiatives focused on increasing the number of children screened, for example: training CHWs on screening for malnutrition, supporting CHWs and ‘*mamans lumières’* (mothers who have been identified as positive deviants for health of their children and have been trained to mentor their peers in behavioural change) in active screening at the community level and a statement read in church after a period of stock-outs. There were also initiatives in favour of the management of inputs, such as active screening sessions if inputs were about to expire or borrowing CSB from another HC. There were also initiatives targeted at infrastructure (construction/rehabilitation of premises for nutritional services) as well as human resources (interruption of holidays of the staff assigned to nutritional services on busy days). The logbooks of two HCs mention rental of a vehicle/motorcycle for distribution and screening in remote sites.

In several logbooks where initiatives on infrastructures and support to active screening were mentioned, HWs have also reported barriers to the implementation to the strategies they had considered. Most managers have reported the lack of resources as having hindered initiatives directed towards support of active screening, as phrased below:

“*We wanted to rent a motorbike for the active detection of malnutrition cases*, *but it was impossible because we did not have financial resources*.*”* (1702L)

All managers who mentioned an initiative for infrastructure also complained about the lengthy administrative procedures. This issue was also repeatedly mentioned by our respondents.

“*No*, *there is a limit not to be exceeded*, *we were told that for expenses exceeding the amount of (*…*) you must first apply for authorisation from the District Office (*…*)*. *yes*, *even if the action plan is validated no one should exceed the amount*, *we are told to write a letter and wait for the answer*.*”* (HC manager 602I6)

One HC reported payment delays as a brake on initiatives to improve nutritional services.

“*They delayed to pay the subsidies of nutrition indicators*, *so no initiatives*.*”* (901I7)

We have also discussed the issue of human resources with our key informants. Human capacities came back several times. An issue is that malnutrition management is often assigned to a single person with some knowledge on nutrition.

“… *yes*, *it's not everyone who is knowledgeable about nutrition services activities*. *As we said*, *too little training is provided*. *Furthermore*, *there is also not enough oversight for these services*. *I think that's why there are no initiatives because there is not enough staff retraining*. *If a nurse gets sick and the other is on holiday*, *the service is not functional*. *I would suggest that the whole HC staff get trained*. *This will ensure that even if one nurse is absent*, *the service will be ensured*.*”* (In-charge of Nutritional services 601C2)

The general low level of technical capacities for nutrition management at the facility level is indeed a constraint. We are far from the principle that malnutrition should be a general concern for all staff in contact with children. In another paper, using the baseline data, we have documented that staff in charge of paediatric consultation in HC in Burundi do not know how to manage child illnesses in an integrated manner; this certainly leads to some missed detection of malnutrition cases [41]. Our end line confirmed that the PBF-N has not modified this situation [36].

In section 3, we formulate the hypothesis that HCs are actually aware of their technical shortcomings and have managed to circumvent their limitations in a smart way.

***Our assessment***: this track was not activated by the PBF-N. Any initiative taken by the HCs stems from pre-existing entrepreneurial capacities, possibly developed under the PBF-FHC.

#### The supervision track

The ‘supervision track’ refers to the possibility that the PBF-N could induce an intensification of the supervision provided by the HDMT and, thus, generate some effects on service delivery. This track obviously partly rests on the effectiveness of the supervision itself. The greater intensity and effectiveness of supervision may come from a greater pro-activity by the HC (e.g. they more eagerly consult supervisors to solve problems with their nutrition services) or from supervisors themselves (e.g. because they see the PBF-N as a leverage for better nutrition services or they want to sharpen their own skills to prepare for the following scale-up).

An obvious key condition for this track to deliver its effects is that health facilities actually receive supportive supervision. This was obviously the expectation of HC staff:

“… *yes for any supervision that is done*, *we expect it to be done in order to improve our services because when the supervisor arrives and finds that some things are not done correctly*, *he shows you how to improve; for things that are very well done*, *you get encouragement to move forward*.*”* (HC manager101I4)

However, according to the results of the end line surveys from managers of HC nutrition services, supervision visits from the HDMT on the subject of malnutrition were not that frequent. Only 42% of HCs in the intervention group (35% of HCs in the control group) reported having undergone a supervision on malnutrition by the district team within the last quarter.

Besides, it is important that supervisors are knowledgeable, credible and empathic. However, according to the qualitative interviews, one of the obstacles for providing more frequent supervision visits was the HDMT's ability to properly carry out supervision of nutrition services.

“*I would say that they themselves have limited capacity to do the supervision*. *Indeed*, *coming as a member of the HDMT to supervise aspects that you do not master properly poses a problem; yes*, *if you do not have a good command on the area you have to supervise it is a problem*. *It is necessary that the supervisors have first and foremost sufficient knowledge on the subject to supervise*.*”* (In-charge of Nutritional services 602I6)

This was confirmed by a HDMT member, who also raised the issue of a lack of resources to allow the person trained on malnutrition to perform more supervision visits alongside the integrated supervisions:

“*It is true that I am trained in nutrition*, *but it happens sometimes that the HC is supervised by a team made of supervisors without any knowledge of malnutrition; in such situations*, *nutritional services are not supervised at all*. *But if there were additional means*, *the supervisor trained in nutrition could revisit these HCs apart from the integrated supervisions that are performed at a bi-monthly basis*.*”* (HDMT member 1402)

The shortcomings of HDMT supervision (irregularity, insufficient integration) are also reported in other documents of the MoH, including the *CTN-FBP* annual reports.

***Our assessment***: this track was not activated during the PBF-N.

#### The health system track

The ‘health system’ track acknowledges that part of the effect of the intervention may come from specific actions taken by health system actors. This would be the case for instance, if the ‘owner’ of the intervention (e.g. the MoH, the World Bank) implement supportive measures to protect their intervention or the impact evaluation.

The whole story of this intervention (see above) indicates that one should not overestimate this effect; a lot of the problems encountered by the PBF-N actually occurred at a system level. Neither the intervention nor the impact evaluation were sufficient arguments to prevent the main supplier of dietary inputs for MAM, WFP, to pull out from a large number of provinces a few months before the start of the intervention. Similarly, despite the obvious shock it would create for the intervention itself, *PRONIANUT* decided to drop the option of local purchase by HCs. Its own solution eventually came very late and proved to be unreliable. Actually, for dietary inputs, the health system never managed to respond to the demand placed by the intervention. At the time of the 2017 end line survey, dietary treatments for MAM were available in only 10 of the 90 HCs, and in the three previous months, there had been stock-outs in 83 HCs (92%).

This constraint is frequently reported in the logbooks and was confirmed by interviews with providers:

“*Yes*, *we had constraints*. *When the intervention started*, *we thought there would be continuity of the program*. *But it happened that for two months or more*, *we worked very little compared to what was expected… If there are stockouts after the start*, *the beneficiaries lose trust in the service*. *Ourselves*, *we saw the continuity undermined by the low reliability of the input suppliers*. *So*, *we were making steps backwards and it was difficult for us to have ‘cured cases’*.*”* (In-charge of Nutritional services 602I6)

It also seems that the system failed to solve other bottlenecks. According to the results from the 2017 survey, the equipment for screening and management of malnutrition was complete in only two (4%) HCs of the control group and in three (7%) HCs of the intervention group. This is a failure of the system track, but possibly also of the culture track. Indeed, as pointed by a health district management team member, this raises also questions about some habits or expectations:

“*I think that at the moment*, *even if they had money*, *they gave priority to other expenses*, *because needs were numerous*. *Yes*, *they did not buy any equipment*. *Having said that*, *it is not easy to find a measuring board or a scale locally*. *Furthermore*, *they were used to receiving them as donations*, *yes it was always donations*!” (HDMT member 1602)

All these problems do not mean that this track was not activated at all. As reported above, we have seen that when food supplements eventually became available, intervention HCs were prioritised over control HCs, as the latter accessed the flours three months later. Intervention HCs also received their first payment three months earlier than the control group.

***Our assessment***: this track was activated, but with too low intensity throughout the intervention to lead to any significant effect.

### How did PBF-N still have some effects?

From the findings presented in our sections 1 and 2, it emerges that six constraints have weighted upon the intervention: (i) initial low skills of HWs; (ii) unavailability of resources (including nutritional dietary inputs and equipment); (iii) payment issues; (iv) suboptimal information; (v) restrictions on autonomy; and (vi) low intensity of supervision. These constraints have weighted upon all HCs and have affected the intensity of the PBF-N, especially during the first year of intervention.

Despite these harsh conditions, the PBF-N had still a significant positive effect on two outputs (number of MAM and number of SAM) and three outcome indicators (MAM recovery rate and duration of treatment both for MAM and SAM) (Table 1). How can we explain this surprising result? We propose to proceed step by step, as below.

The first step is to exclude the ‘gaming’ scenario, under which the effects would be fake and the result of opportunistic or fraudulent behaviours by HCs (e.g. invention of cases, enrolment of children not malnourished). Burundi has built a strong verification system into its PBF program. This verification system is strict (with a low threshold for penalties), regularly audited and has been assessed as very reliable [45]. We can exclude the hypothesis of ghost patients. We can also reject cheating with the outcome. As reported in Table 1 (indicator 11), from the baseline to the end line, the increase in the proportion of ‘false positives’ in the intervention HCs was very limited and non-significant. Actually, intervention HCs missed a lot of malnourished children (indicator 12)–a result consistent with our observation that insufficient effort was given to screening by clinical staff.

We can now move to step 2: can we attribute the effects to some specific tracks? We know we can exclude a contribution from the three tracks which were not activated (‘culture transformation’, ‘better supervision’ and ‘support from the system’). The explanation must come from the others: the income, the cash, the incentive and the information tracks.

As a reminder, our research design set some specific conditions for the control group: they also received some complementary funding during the intervention and this money arrived into their bank account. The match was not perfect and there was a slight delay for the control group, but our own interpretation is that any significant effects reported in Table 1 do not stem from the extra income received in cash. This indicates that the effect for the two output and three outcome indicators may only come from the two other tracks: the incentive and information tracks. This is consistent with the fact that those five indicators were also rewarded by the PBF-N scheme (the first two under the quantitative component of the PBF-N, and the three others under the quality checklist) (see S1 File): the staff knew that these indicators matter for good nutrition services and that they would be rewarded if they improve them.

Still, this does not tell us how they managed to increase the volume and improve the quality of care despite the many constraints.

How did the intervention HCs manage to increase their number of MAM and SAM cases? This firstly required HCs to detect more malnourished children. We know that, according to the guidelines, HCs could do that through two strategies: during growth monitoring sessions and during paediatric curative consultations with the Integrated Management of Child Illness (IMCI) strategy. However, thanks to the baseline and end line facility surveys, we know that nothing happened at that level: for these two strategies, HCs were weak at the start and still weak at the end line (indicator 9 in Table 1). This suggests that HCs found another strategy. Our qualitative data (cf. initiatives reported under the culture track) provides the best explanation: for their recruitment of patients, HCs have relied on the CHWs and “*mamans lumières”*. Actually, going for a community-based recruitment strategy instead of a HC-based approach makes a lot of sense given the limited skills [41] and capacities (Table 2) at the HC level. Furthermore, mobilizing the CHWs of the catchment area was also logical as in the intervention group, CHWs were rewarded by the PBF-N. Then, the screened children were sent directly to nutritional services on the opening days of these services.

The active screening of malnutrition at the community level and referral of cases to the HCs by the CHWs are frequently mentioned in several logbooks of the intervention group and were confirmed by several respondents. For instance:

“*With regards to the observation that nothing has changed in curative consultation*, *the explanation is that wherever they came from*, *mothers were aware of the existence of the nutrition service*. *The CHWs had given them an orientation*. *They were arriving at the HC knowing the service in which they had to go*. *Thus*, *they did not go to curative consultation but went directly into the nutritional service*. *Parameters (of the children) were taken there*. *The (positive) tested ones were then taken care of directly in this service*. *This is why there is no trace of them in the curative consultation records*.*”* (In-charge of Nutritional service 902I1)

This result is consistent with our quantitative data. Extra analyses, combining the community and facility databases, have shown that a significantly higher proportion of children have been managed for their malnutrition status in communities served by a HC staffed with a health worker dedicated to health promotion and supervision of CHWs. [37].

The significant shortening of the duration of treatment and the higher recovery rate for SAM constitutes our second puzzle. How was this possible, given the limited availability of food supplements and the difficulties encountered in the purchase of these supplements by the HCs?

Our qualitative data suggest that HCs have reacted to these constraints by improving the management of these supplements at the two levels under their control: at the facility level and at the level of the patients themselves. Several respondents explained to us that under the new incentive structure, they approach their stock management in a different way.

“*With WFP*, *it was donations*. *In fact*, *what you get without effort you use it without precautions*. *For example*, *in the past*, *it happened that we were a bit careless in our handling of the inputs*. *This caused losses*. *But when it's the HC that has bought the products*, *we pay more attention*: *we know we are losing our own money*. *Staff of the HC realised that the CSB was expensive and became sensitive to its good management*. *With the WFP CSB*, *there were some who did not even know where it came from*. *When we buy it on our own*, *everyone makes sure there is no loss*.*”* (In-charge of Nutritional services 602I6)

“*We asked the CHWs to ensure*, *for each child already admitted to the program*, *that the nutritional supplements are not sold or shared with other children*. *Indeed*, *it often happens that a mother who receives nutritional supplements for a malnourished child has other children; she may be tempted to share the ration with her other children*.*”* (HC manager 101I4)

### Updating the ToC

**Fig 3:**
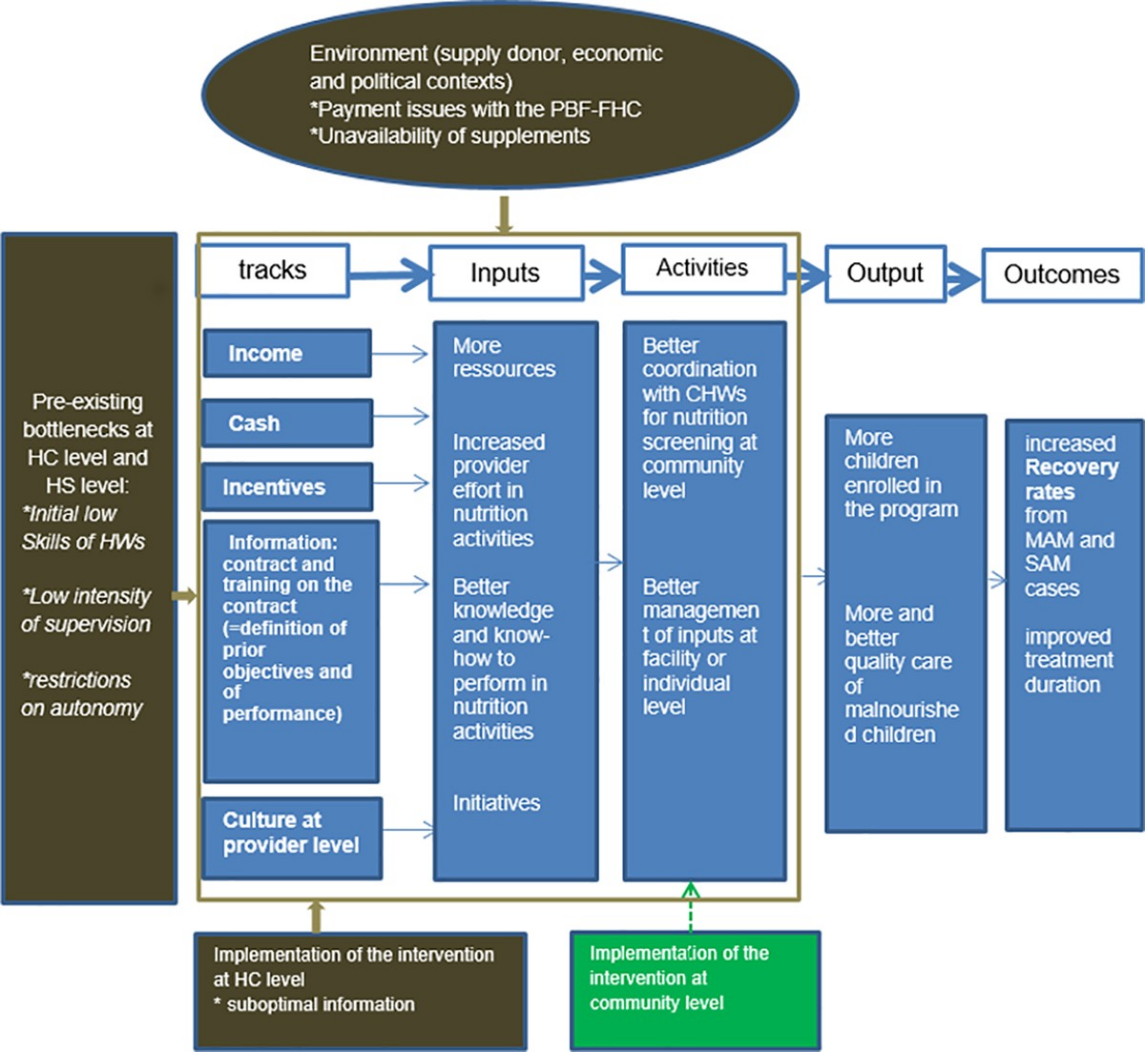
Updated ToC of the PBF-N.

Based on our empirical work, it is now possible to revise our ToC. As already said above, despite many constraints, HCs have managed to improve their performance. In our updated ToC (Fig 3), we removed two tracks for which we did not find evidence of a contribution (supervision and health system). Although not activated by the present intervention, we have kept the ‘culture at provider level’ track. Indeed, it is thanks to the entrepreneurial ethos promoted by PBF that HCs have taken initiatives which led to a better performance. Similarly, we have kept the income and cash tracks: without them, the PBF-N would have ‘shot blank’. To lead to better outputs (column 4) and outcomes (column 5), HC staff have disregarded most of the activities we had anticipated in our initial ToC (Fig 1): instead, they made the choice to better coordinate with the CHWs and to better manage inputs at the facility or individual level. The actions with the CHWs have been facilitated by the implementation of the PBF-N at the community level–a component of the intervention we overlooked in our original ToC. In Fig 3, we have also mapped several external factors which have negatively impacted the intervention.

## Discussion

In this study, we have tried to better understand the actual tracks through which a PBF scheme delivers its effects. Our specific case was the pilot integration of nutrition services within the PBF-FHC scheme in Burundi. We have considered seven tracks, and, through a multi-methods approach, we have been able to rule out some of these.

A picture emerged of HC staff demonstrating agency. Constraints were tight, yet, HCs from the intervention group found smart ways to circumvent them and to improve the performance of their nutrition services. As evidenced more extensively by the impact evaluation [36], they have been selective in their effort. Firstly, they did not bother with many aspects which might be seen as important from a programmatic perspective (e.g. more equipment, growth monitoring in curative consultation, IMCI, etc), either because they were not aware of them or by choice (see Table 1). Secondly, they also decided not to invest in one activity rewarded by the PBF-N (growth monitoring in curative consultation), which suggests that they have analysed in the information provided by the scheme. Instead, they relied on CHWs and at the facility level, focused on tasks and outcomes which were both (i) valued from the perspective of the PBF-N and (ii) achievable at a limited cost. All this suggests an optimisation behaviour with a selective responsiveness to the information and incentive tracks.

Beyond this specific finding, we believe that this study contributes to three broader matters of interest: the ToC of PBF and its study, PBF in FCAS and the application of PBF to nutrition services. We will discuss these matters in this order.

As in several recent contributions [46–49], our study adopted a broad view on the ToC of PBF. Our case shows that the intervention itself (PBF-N) interplayed with at least three other pre-existing interventions, each having its own ToC: delivery of nutrition services at the HC level, supervision by the district team and PBF-FHC. The effects of PBF-N have been partly determined by problems at the level of these other interventions. This dependence upon the whole health system has been documented in many PBF studies; barriers vary across experiences, but several of those reported in this study (competencies of human resources at the facility level, skills of supervisors, supply of inputs and delays in payment) have been reported in other experiences [50–60]. Such a dependence confirms the systemic nature of PBF [61], but at short term, it has an impact on the effectiveness of the strategy. In our opinion, we must accept this complex reality. The PBF-N might have had more effect with some 'twists' (for example, higher fees for the nutrition services or ear-marking), but because the intervention was part of a broader policy, it has respected certain rules. Our case study shows that patience pays: several problems have gradually been solved by decentralised actors themselves. Although things did not happen as expected, the central tenet of PBF (once incentives are right, one can rely on the agency of actors) has played out. Conversely, the problems observed with the centralised procurement of CSB do not plead in favour of solutions brought from the top (a model of which PBF experts are critical).

Our study confirms that the PBF ToC is complex. Our approach with the tracks, which is one approach among others, consolidates the case in favour of mixed methods design, which allow a deeper and broader understanding of the phenomena under study [62].

Context has played an important role in the story of PBF-N. This observation is not atypical for an intervention in a FCAS and has been documented in other studies on PBF in such settings [16,63–65].

By definition [66], a fragile setting is prone to deterioration; this is exactly what happened in Burundi in 2015. Our study has shown that this does not make things simpler, neither for the intervention nor for its study. A recent study [67] identified challenges in conducting health system research in FCAS. We experienced several of those challenges, including the shock waves from the context, a limited access to the field during the tensest period of the political crisis and lack of a strong supportive ecosystem for the cluster-randomised controlled trial. Our capacity to extract lessons confirms that mixed methods research designs are particularly suited for such adversarial settings.

In general, we need more health system research in FCAS. Indeed, FCAS are also the places where the needs for improvements are the greatest. This is particularly true for child malnutrition. The experience reported in this study reminds us that child malnutrition is not easy to address. The effects of PBF-N were positive, but probably not as great as expected (see indicators 6 and 7 in Table 1) [37]. Some of the constraints are probably specific to Burundi. Both the limited capacities for malnutrition management at district and facility level and the difficulties experienced with the local purchasing of the nutrition inputs may be the heritage of the reliance, in recent decades, upon humanitarian actors. In more stable LICs, one can hope that these capacities are stronger. Furthermore, one must appreciate the policy dynamics such interventions and studies trigger. PBF-N contributed, for instance, towards convincing the World Bank that it had to invest against malnutrition in Burundi in a more holistic manner. As for the lessons beyond Burundi, our study supports the idea of integrating the management of malnutrition into PBF programs, both at HC and community levels, but it also calls for sufficient attention to capacities at different levels of the health system.

Our study has several limitations. We could analyse 75 logbooks, out of the 90 HCs involved in the impact evaluation. Our interviews covered a sub-sample of HCs and district supervisors. Because of that, we cannot exclude that we missed some other explanations for the effects or other observed phenomena. As reported in the results section, for some ‘tracks’ (e.g. culture), we did not collect good variables with our quantitative survey instruments. To generate the most comprehensive understanding of the ToC of the PBF-N scheme, we used different techniques; these have allowed us to triangulate information. However, we cannot exclude that, like in any qualitative social research, respondents sometimes gave the answers that they thought we expected. Our close follow-up of the filling of logbooks and our own conduct of carrying out the interviews (1^st^ author) must have limited these risks.

There are several directions for future research. In Burundi, it would be interesting to examine what happened after the pilot intervention, for example: have the contextual constraints been relaxed; how has this evolution (if any) affected the performance at the HC level; and what are the consequences of the decision by the *CTN-FBP* to keep SAM only for the scale-up? It would also be interesting to have more qualitative and quantitative research on the contribution of CHWs towards the national fight against malnutrition. Beyond Burundi, we need more evidence on applications of PBF to malnutrition management, across different types of settings. We also believe that more empirical studies on PBF ToC are still needed. We hope that this study can inspire other researchers.

## Conclusion

In this study, we have investigated the constraints that have affected the effectiveness of the extension of the PBF policy to focus on malnutrition in Burundi. We have proposed some explanations of how, despite these constraints, the intervention has had an effect. Positive changes occurred at the level of variables on which the frontline actors had control. Getting incentives right at decentralised level is fine, but our study also shows that it won’t be enough to resolve child malnutrition in Burundi. This problem, central to the development of the country and the well-being of its youngest population, calls for a massive effort at many levels of society. As far as the health system is concerned, pro-active steps should be taken for staff competence, supervision by district teams and supply of food supplements. We also believe that donors should sustain their effort in the sector. This chronic crisis will not be solved by one single intervention.

### Ethical approval

This study builds on a larger impact evaluation, whose protocol was approved by the *Comité National d’Ethique pour la protection des êtres humains participants à la recherche biomédicale et comportementale du Burundi* (decision on 28/4/2014) and the ethical committee of the University of Antwerp (Belgian registration number: B300201421689; UZA ethics committee 14/22/240). In addition, the study has been approved by the Institutional Review Board of the Institute of Tropical Medicine (IRB #951/14).

Written informed consent was obtained from health centre managers for logbooks filling in. Written informed consent was also obtained from Health Centre staff and the Health District Management Team interviewed. Participants were allocated codes and no identifying information was recorded.

## Supporting information

S1 File Electronic annex. (DOCX)
