# Supplementary material for: Effects in spite of tough constraints - A theory of change based investigation of contextual and implementation factors affecting the results of a performance based financing scheme extended to malnutrition in Burundi
Source: PLoS One. 2020 Jan 13;15(1):e0226376. doi: 10.1371/journal.pone.0226376 (PMC6957191; doi:10.1371/journal.pone.0226376)
Supplement: S1 File — (DOCX) [file pone.0226376.s001.docx]

**Electronic Annex: measures, reward structure and rating criteria applied to HCs by the PBF-N**

The PBF-N combined a quantitative and a qualitative measurement of the performance of nutritional services by HCs.

**Quantitative component of performance**

The quantitative measurement (‘PBF-N subsidies’) focused on four services: (1) the management of MAM (Table 1:1), (2) the management of SAM (Table 1:2), (3) diagnosis and referral of malnourished children (Table 1:3) and (4) growth monitoring (Table 1:4).

Among these services, the first two were the most important: these are the services most likely to generate a direct health benefit. In fact, only HCs with functional MAM and SAM services were eligible for the impact evaluation. The ‘diagnosis and referral’ indicator only applies to HCs without a capacity to manage malnourished children. It also turned out to be a fallback position for HCs with a management capacity in case of stockouts of nutritional inputs.

**Table 1: quantitative measures of performance**

| **Indicators** | **Criteria to be considered** | **Source of verification** | **Fee** |
| --- | --- | --- | --- |
| 1. Number of children under five years diagnosed with Moderate Acute Malnutrition (MAM) and correctly managed according to national protocol and who have completed treatment during the month | Definition of MAM:  -3 SD≤W/H<-2 SD  Or  115 mm ≤MUAC <125 mm (if H>65cm) and  absence of oedema | MAM register  Individual files | 3000 BIF (2015)  47000 BIF (2016) |
| 2. Number of children under five years diagnosed with Severe Acute Malnutrition (SAM) and correctly managed according to national protocol and who have completed treatment during the month | Definition of SAM:  W/H<-3SD; or  MUAC<115 mm; Or  Bilateral pitting oedema and absence of medical complications^[[1]](#footnote-1)^ | SAM register  Individual files | 2500 BIF (for the whole period of the intervention) |
| 3. Number of children under five diagnosed with acute malnutrition (moderate and/or severe), referred to and arrived at a HC with MAM or SAM capacity | See MAM and SAM criteria | *Referral and counter referral forms *Register of cases referred at HC level with MAM and SAM | 700 BIF |
| 4. Number of children under two, whose weight and height were measured during the month and whose weight/age growth curve follows a *good* growth trajectory | The weight/age curve must be ascending that is, curve does not mark a decrease in weight for more than two measurements and/or if the curve shows such a decrease in weight, that the curve shows a return towards the initial trajectory and/or shows a catch-up of weight) | Notebook/health cards with growth curve for boys and girls | 700 BIF |

For these four ‘indicators’, verification was done on a monthly basis as for the PBF-FHC scheme.

The fee for the MAM service has varied. The 2016 amount includes the cost of purchasing inputs as a result of WFP's decision to stop supplying inputs in most provinces. The input cost per child is estimated at 90% of the amount allocated per validated child.

**Qualitative component of performance**

The qualitative measurement of the performance rested on a checklist documenting different dimensions of the nutritional services. The assessment was carried out on a quarterly basis, as for the other quality ‘indicators’ of the PBF-FHC scheme. The PBF-N checklist consisted of two groups of qualitative ‘indicators’: those related to growth monitoring and those related to MAM and SAM services.

**Table 2: qualitative ‘indicators’ related to growth monitoring (39% of the checklist):**

| **Quality dimension under evaluation (input, process, outcome)** | **Rules for getting the points** | **Maximum number of points available** |
| --- | --- | --- |
| 1. Availability and functionality of equipment/tools required for growth monitoring (inputs) | Visit of the nutrition ward and visual observation of the availability of items:   1. Measuring rod: Yes / No 2. Scale: Yes / No 3. Infant surveillance register: Yes /No 4. Infant individual files: Yes / No 5. Mother and child booklets: Yes / No   Max Score: 20 pts  Unmet criterion = - 5pt | 20 pts |
| 2. Outcome for the children under two years old who attended a growth monitoring and promotion session (no malnutrition) | Review of all patient files of children under two and calculation of the proportion of children whose weight for height ratio is greater than -2SD:  If less than 95%: 0 pts  If between 95 & 97%: 30 pts  If higher than 97%: 50 pts | 50 pts |
| 3. Outcome for the children under two years in the catchment area of the HC who attended the growth monitoring and promotion sessions | At least 90% of the children under two living in the catchment area of the HC have attended the growth monitoring and promotion sessions over the last three months: 30pts | 30 pts |
| 4. Quality of the individual session (process) | Review all patient files of the last quarter and check if for each individual session/visit, the following advice has been provided:  (1) discussion of current weight and height of the child: Yes / No  (2) ask the mother if she has any concerns about the child’s weight or eating habits: Yes / No  (3) provision of nutrition and development advice, according to age of the child: Yes / No  A criterion met= 10pts  Criterion not met= 0pt | 30 pts |
| 5.The growth curves W/A and H/A are well traced (process) | Review all patient files of the last quarter and check whether the weight and height used to plot the growth curves correspond to those reported in the infant register.  If ≥ 90%: 10 pts | 10 pts |
|  | **TOTAL** | **150 pts** |

**Table 3: qualitative indicators for MAM services (30% of the checklist)**

| **Quality dimension under evaluation (input, process, outcome)** | **Rules for getting the points** | **Maximum number of points available** |
| --- | --- | --- |
| 1. Inventory of equipment for the MAM service exists and updated (inputs) | Visit of the MAM service and visual observation of the equipment inventories of the service:  If an initial inventory exists= 10 pts  If an initial inventory missing & not updated= 0 | 10 Pts |
| 2. Availability and functionality of equipment/tools required for screening and management (inputs) | Visit of the MAM service and visual observation of the availability of items:   1. Measuring rod: Yes / No 2. MUAC: Yes / No 3. Scale: Yes / No 4. Admission and exit criteria (displayed): Yes / No 5. MAM register: Yes / No 6. National protocol of acute malnutrition management: Yes / No 7. W/H tables   max score= 20pts  Criterion not met= -5pts | 20 Pts |
| 3. Quality of MAM management: compliance with admission criteria (process) | Review of 10 random patient files and identification of those for whom the admission criteria are in line with the guidelines of the MAM management manual.  If compliance with criteria for more 9 cases= 10pts,  If compliance with criteria for less than 8 cases= 0pts | 10 Pts |
| 4. Quality of MAM management: Management register correctly filled in (process) | Review of the infant register for MAM service and check if, for each patient, all the columns of the register are filled in.  Criterion met= 10pts | 10 Pts |
| 5. Quality of MAM management: Correct case management of 20 cases (process) | Visit of the MAM service and review of the register and individual files of 20 random patients. For each case, check if:   1. Patient identification exists: Yes / No 2. Criteria and symptoms at admission are mentioned: Yes / No 3. Management is completed according to the protocol: Yes / No 4. Managed by a nurse trained on the protocol of acute malnutrition management: Yes / No   5pts per criterion | 20pts |
| 6. Quality of MAM management: compliance with performance indicators (outcomes) | Review of the monthly reports (of the last quarter) of the MAM service and check the performance indicators achieved by the HC. For each report, check if:   1. Average weight gain (2-3g/kg/day): Yes / No 2. Average length of stay (<2 months): Yes / No 3. Recovery rate (>75%): Yes / No 4. Death rate (<3%): Yes / No 5. Dropout rate (<15%): Yes / No 6. Non-respondent rate (<10%): Yes / No   All criteria met= 30pts  1 missing criterion= 0pt | 30 Pts |
| 7. Availability of required dietary inputs and drugs for systematic treatment (inputs) | Visit of the MAM service and visual observation of the availability of items:   1. CSB/UNIMIX: Yes / No 2. Vitamin A: Yes / No 3. Folic acid: Yes / No 4. Albendazole/Mebendazole: Yes / No 5. Amoxicillin: Yes / No 6. Artemisinin-based Combination Therapies (ACTs): Yes / No   All inputs available= 10pts  1 missing input= 0pt | 10pts |
|  | | **110 pts** |

**Table 4: qualitative indicators for SAM services (31% of the checklist)**

| **Quality dimension under evaluation (input, process, outcome)** | **Rules for getting the points** | **Maximum number of points available** |
| --- | --- | --- |
| 1. Inventory of equipment for the SAM service exists and updated (inputs) | Visit of the SAM service and visual observation of the inventories of equipment of the service:  If an initial inventory exists= 10 pts  If an initial inventory missing & not updated= 0pts | 10 pts |
| 2. Availability and functionality of equipment/tools required for screening and management (inputs) | Visit of the MAM service and visual observation of the availability of items:   1. Measuring rod: Yes / No 2. MUAC: Yes / No 3. Scale: Yes / No 4. Admission and exit criteria (displayed): Yes / No 5. SAM register: Yes / No 6. National protocol of acute malnutrition management: Yes / No 7. W/H tables: Yes / No   Max score = 20pts  Criterion not met = -5pts | 20 pts |
| 3. Quality of SAM management: compliance with admission criteria (process) | Review of 10 random patient files and identification of those for which the admission criteria are in line with the guidelines of the SAM management manual.  If compliance with criteria for more than 9 cases= 10ts  If compliance with criteria for less than 8 cases= 0pts | 10 pts |
| 4. Quality of SAM management: management register correctly filled out (process) | Review of the infant register for SAM service and check whether, for each patient:   1. the SAM Number is mentioned: Yes / No 2. all the columns of the register are filled in: Yes / No   Both criteria met= 10pts  1 missing criterion= 0pts | 10 pts |
| 5. Quality of SAM management: correct case management of 20 cases (process) | Visit of the SAM service and review of the register and individual files for 20 random patients. For each case, check if:   1. Patient identification exists: Yes / No 2. Criteria and symptoms at admission are mentioned: Yes / No 3. Assessment of medical complications & lack of appetite: Yes / No 4. Diagnosis is mentioned: Yes / No 5. Management is done according to the protocol: Yes / No 6. Managed by a nurse trained in the protocol of acute malnutrition management: Yes / No   5 pts per criterion | 30pts |
| 6. Quality of SAM management: compliance with performance indicators (outcome) | Review of the monthly reports (of the last quarter) of the MAM service and check the performance indicators achieved by the HC. For each report check if:   1. Average weight gain (4-6g/kg/day): Yes / No 2. Average length of stay (30days): Yes / No 3. Recovery rate (>75%): Yes / No 4. Death rate (<5%): Yes / No 5. Dropout rate (<15%): Yes / No 6. Non-Respondent Rate (NRR) (<10%): Yes / No   All criteria met= 30pts  1 missing criterion= 0pts | 30pts |
| 7. Availability of required dietary inputs and drugs for systematic treatment (inputs) | Visit of the SAM service and visual observation of the availability of items:   1. Plumpy-Nut: Yes / No 2. Vitamin A: Yes / No 3. Folic acid: Yes / No 4. Albendazole/Mebendazole: Yes / No 5. Amoxicillin: Yes / No 6. Artemisinin-based Combination Therapies (ACTs): Yes / No   All inputs available= 10pts  1 missing input= 0pts | 10pts |
|  | | **120 pts** |

Overall (Tables 2,3 & 4), the results from this qualitative assessment were used to determine a quality bonus or a penalty, according to the following criteria:

- **≥ 80%: quality bonus = quality score x 25% x Subsidies of PBF-N of the previous quarter**
- **60 to 79.9%: no quality bonus**
- **50 to 59.9%: - 10% of PBF-N subsidies of the previous quarter**
- **30 to 49.9%: - 20% of PBF-N subsidies of the previous quarter**
- **< 30%: - 25% of PBF-N subsidies of the previous quarter**

1. Cases of medical complications must be referred to the hospital. [↑](#footnote-ref-1)
